# The Flora Compositions of Nitrogen-Fixing Bacteria and the Differential Expression of *nif*H Gene in *Pennisetum giganteum* z.x.lin Roots

**DOI:** 10.1155/2021/5568845

**Published:** 2021-04-23

**Authors:** Biaosheng Lin, Jiamin Liu, Xue Zhang, Changren Weng, Zhanxi Lin

**Affiliations:** ^1^College of Life Science, Longyan University, Longyan 364012, China; ^2^Key Laboratory of Fujian Universities Preventive Veterinary Medicine and Biotechnology, Longyan University, Longyan 364012, China; ^3^China National Engineering Research Center of JUNCAO Technology, Fujian Agriculture and Forestry University, Fuzhou 350002, China

## Abstract

The flora compositions of nitrogen-fixing bacteria in roots of *Pennisetum giganteum* z.x.lin at different growth stages and the expression and copy number of nitrogen-fixing gene *nif*H were studied by Illumina Miseq second-generation sequencing technology and qRT-PCR. The results showed that there were more than 40,000~50,000 effective sequences in 5 samples from the roots of *P. giganteum*, with Proteobacteria and Cyanobacteria as the dominant nitrogen-fixing bacteria based on the OTU species annotations for each sample and *Bradyrhizobium* as the core bacterial genera. The relative expression and quantitative change of *nif*H gene in roots of *P. giganteum* at different growth stages were consistent with the changes in the flora compositions of nitrogen-fixing microbia. Both revealed a changing trend with an initial increase and a sequential decrease, as well as changing order as jointing stage>maturation stage>tillering stage>seedling stage>dying stage. The relative expression and copy number of nifH gene were different in different growth stages, and the difference among groups basically reached a significant level (*p* < 0.05). The relative expression and copy number of *nif*H gene at the jointing stage were the highest, and the 2^-△△CT^ value was 4.43 folds higher than that at the seedling stage, with a copy number of 1.32 × 10^7^/g. While at the dying stage, it was the lowest, and the 2^-△△CT^ value was 0.67 folds, with a copy number of 0.31 × 10^7^/g.

## 1. Introduction

Nitrogen is one of the most important basic elements in living organisms and an important influencing factor for plant growth and development. Biological nitrogen fixation is an important way for many plants to obtain nitrogen nutrition. Due to its economical and pollution-free characteristics, biological nitrogen fixation is the hot topic in the field of agricultural biotechnology in recent years [1, 2]. The *nif*H (nitrogen fixation gene H) gene is a common gene encoding a nitrogenase-containing ferritin in nitrogen-fixing microorganism. It plays an important role in biological nitrogen fixation process and is the most conserved functional gene among all nitrogen-fixing microorganisms [3]. Cantera et al. [4] have reported that the phylogeny of *nif*H gene is significantly consistent with 16S rRNA, with highly conserved sequences, abundant data information, and variable regions. Therefore, *nif*H gene has become an ideal genetic marker for nitrogen-fixing microorganisms, which can be used to prove the existence of nitrogen-fixing bacteria in samples [5] and to reveal the relationship between the community structure of nitrogen-fixing bacteria and the environment [6]. *Pennisetum giganteum* z.x.lin is introduced from South Africa by the Institute of JUNCAO at Fujian Agriculture and Forestry University from 2005 to 2007. It is a perennial grass, belonging to the genus *Pennisetum*. It has a developed root system, strong drought resistance, and strong ecological control function for windbreak and sand fixation and is an energy and ecological grass with good application potentials [7]. Gramineous plants, especially their roots, are rich in endogenous nitrogen-fixing bacteria. During past many years, researchers have found the resources with abundant endogenous nitrogen-fixing bacteria from roots and leaves of gramineous plants such as *Azospirillum* [8], *Herbaspirillum seropedicae* [9], *Klebsiella* [10], and so on. The^15^N isotope dilution, N natural abundance, and N balance techniques have applied to confirm that some tropical gramineous plants such as *Saccharus* spp. [11], *Leptochloa fuscu* [12], and *Oryza sativa* [13] could obtain partial nitrogen sources for their growth by biological nitrogen fixation. The content of endogenous nitrogen-fixing bacteria is related to nitrogen-fixing efficiency and nitrogen-fixing level of plants. Endogenous nitrogen-fixing bacteria can be used as an important index to measure nitrogen-fixing efficiency of gramineous plants, and the changes in flora community abundance and diversity can indirectly reflect nitrogen cycling status and nitrogen-utilizing efficiency of plants [14]. Therefore, understanding the abundance, diversity, and *nif*H gene expression of endogenous nitrogen-fixing bacteria in plants has great significance for exploring molecular regulation mechanism of nitrogen fixation in plants [15].

High-throughput sequencing technology can determine millions of DNA sequences one time. This technology breaks through the limitation of microbiology based on traditional pure culture, which can conduct quantitative analysis of the samples with noncultivable, dominant, and trace bacteria and can reflect comprehensively and accurately flora compositions and abundance in samples [16]. Real-time quantitative reverse transcription PCR (qRT-PCR) is novel nucleic acid quantitative technique developed by American Applied Biosystems Company in 1996 based on an ordinary PCR qualitative technology. Due to its high efficiency, high sensitivity, and accurate quantification, it has been widely applied in the field of gene expression and analysis in recent years [17, 18]. With the continuous development of molecular biology and its corresponding techniques, high-throughput sequencing technology and qRT-PCR technology have been gradually applied for the detection of *nif*H gene in nitrogen-fixing microorganisms, which can conduct both qualitative and quantitative analysis, thus making it possible to quickly, comprehensively, and accurately detect and analyze the flora compositions and abundance, and the *nif*H gene expression in nitrogen-fixing bacteria in samples.

Therefore, in the present study, the Illumina Miseq second generation sequencing technology and qRT-PCR technology were adopted for analyzing the changes in flora compositions, *nif*H gene expression, and copy number in nitrogen-fixing bacteria from *P. giganteum* at different growth stages, which will uncover the correlations among the growth of *P. giganteum* and flora compositions and *nif*H gene expression in nitrogen-fixing bacteria, further enrich the resources of endogenous nitrogen-fixing bacterial strains in gramineous plants and explore the theoretical basis associated with endogenous nitrogen-fixing bacterial species distribution and change rule, molecular regulatory mechanisms of nitrogen-fixing bacteria, nitrogen metabolism, and its interactions with crops in *P. giganteum* and other gramineous plants.

## 2. Materials and Methods

### 2.1. Reagents and Instruments

Bacterial genomic DNA extraction kit (No. D6942-01) was purchased from OMEGA Biotek Company. Bacterial genomic total RNA extraction kit (No. DP430) was purchased from Tiangen (Beijing) Company. DNA marker, restriction enzymes, reverse transcription reagent, Taq DNA polymerase, and other corresponding reagents were all purchased from TaKaRa Bao Bioengineering (Dalian) Co., Ltd. Competent cell *E. coli* DH5*α* was provided by Beijing ComWin Biotech Co., Ltd. T vector was purchased from TransGen Biotech (Beijing) Company. PCR amplified primers for bacteria *nifH* gene in high-throughput sequencing and *nif*H gene primers for qRT-PCR analysis were all used universal primers for bacterial *nif*H gene, and the primers were synthesized by Beijing Allwegene Technology Co., Ltd.

The main equipment used in this study was PCR amplification apparatus (Model number: ABI-2720), Real-Time PCR apparatus (Model number: ABI 7500), Biological analyzer (Model number: Agilent2100), GEL imaging system (Model number: Agilent2100), Ultraviolet spectrophotometer (NANODROP 2000) and Electrophoresis apparatus (Model number: DYY-6C).

### 2.2. Sample Collection and Processing

From August to December in 2019, the roots at the seedling stage (the period from emergence to the beginning of the first tiller), tillering stage (the period from the beginning of tiller to the beginning of jointing), jointing stage (the period from the beginning of the jointing to the basic stop of the extension), maturation stage (the period from the basic stop of the extension to the beginning of the leaf turning yellow and withering), and dying stage (the period from the beginning of the leaves to turning yellow and withering to the death of the entire plant) of *P. giganteum* planted at the same batch were harvested in an experimental field at Jinshan campus of Fujian Agriculture and Forestry University. The collection site was 700 m above sea level, with an average annual precipitation of 1670 mm, soil pH 7.28 ± 0.05, organic matter 12.53 ± 0.65 g/kg, and total nitrogen 0.63 ± 0.04 g/kg. The samples were collected at multiple sites, and according to the S-type principle, each sample selects five sampling parties with spacing greater than 10 m. The root of the sample was separated from the soil by excavation, and the complete root system of the sample was obtained carefully. The obtained root systems were rinsed with water, mixed, and placed into sterile sealing bags for later use. Totally 5 samples were collected. The samples were routinely disinfected on the aseptic operation table in the laboratory; each sample root was sampled for 10 g, washed with running water for 1 h, soaked in 75% ethanol for 60 s, treated with 2% sodium hypochlorite for 20 min, and washed with aseptic water for 4 times. After the assay was approved, the test samples were used for the analysis of the next high-throughput sequencing and qRT-PCR technology. The data determination of the samples was conducted in triple for obtaining the mean value.

### 2.3. High-Throughput Sequencing and Composition Analysis of Nitrogen-Fixing Bacteria in Each Sample

After aseptic treatment, total DNA of bacteria in each sample was extracted according to OMEGA kit instructions. A fragment of 360 bp was chosen as the PCR amplification fragment of *nif*H gene with nitrogen-fixing function (*Nif*H gene is a specific band of about 360 bp and has a relatively conservative region, so it is often used to identify the existence of nitrogen-fixing microbial strains). The PCR amplification was conducted using the designed primers, PCR reaction system, and reaction conditions according to the methods from Poly Primer sequences are follows [19]: *nif*H-F (Primer F): 5′-AAAGGYGGWATCGGYAARTCCACCAC-3′; *nif*H-R(Primer R): 5′-TTGTTSGCSGCRTACATSGCCATCAT-3′. PCR reaction system (25 *μ*L) is as follows: 5× Reaction buffer 10 *μ*L, 5 × GC buffer 5 *μ*L, dNTPs mixture (2.0 mmol/L) 5 *μ*L, Primer F (10 *μ*M)1 *μ*L, Primer R (10 *μ*M) 1 *μ*L, DNA Template 2 *μ*L, ddH2O 8.75 *μ*L, and Q5 DNA Polymerase (2 U/*μ*L) 0.25 *μ*L. PCR reaction conditions are as follows: 98°C 2 min, 98°C 15 s, 55°C 30 s, 72°C 30 s, 35 cycles, and 72°C 5 min. The amplified product was electrophoresed on a 1% agarose gel and recovered by DNA purification kit, and the target genetic fragments were subjected to sequence determination and analysis by Miseq (Illumina) high-throughput sequencing platform at Beijing Allwegene Technology Co., Ltd. After obtaining Miseq raw data, the original sequencing data of both ends were subjected to the treatments of eliminating impure data, splicing, and chimeric sequencing. The high-quality sequences were subjected to the treatment by CD-HIT (Cluster Database at High Identity with Tolerance) classification method, and the sequences with the similarity of greater than or equal to 97% were classified as an OTU (operational taxonomic units) unit. The sequences with largest abundance according to OTU classification were listed as the representative sequences [20], and the compositions of nitrogen-fixing bacteria in each sample were analyzed on the basis of representative sequences acquired according to the sequence analysis of the sample group. In addition, according to the abundant information of each sample at the genus level, PCA analysis (principal component analysis) was carried out for samples by the R software (the R project for statistical computing), and the similarity among sample groups was compared.

### 2.4. Extraction of Total RNA from Samples and the Analysis of nifH Gene Expression

The relative quantification of *nif*H gene in each sample was determined by qRT-PCR. Total RNA in each sample was extracted by corresponding extraction reagents according the manufacturer's instructions. The integrity of the extracted samples was evaluated by 1.2% agarose gel electrophoresis, and the concentrations of extracted RNA were determined by ultraviolet spectrophotometer at OD_260/280_. Totally 1.0 *μ*g of total RNA after quality evaluation was used for the reverse transcription to generate cDNA according to the operation instructions of the kit. Total volume of the reverse transcription system was 20 *μ*L, which included 4.0 *μ*L of Mater Mix (containing 2.0 *μ*L of 5× gDNA Eraser buffer, 1.0 *μ*L of gDNA Eraser, and 1.0 *μ*g of Total RNA), 1.0 *μ*L of PrimeScript RT Enzyme Mix, 1.0 *μ*L of RT Primer Mix, 4.0 *μ*L of 5× PrimeScript Buffer, and 10.0 *μ*L of RNase-free H_2_O. Two pairs of primers for *nif*H (target gene) and 16S rRNA (internal reference) were designed for PCR using the reverse transcription products of each sample as the templates. Primer sequences are follows: *nif*H-F (Primer F), 5′-AAAGGYGGWATCGGYAARTCCACCA C-3′; *nif*H-R (Primer R), 5′-TTGTTSGCSGCRTACATSGCCATCAT-3′; 16S-F (Primer F), 5′-CCTACGGGTSGCAGCAG-3′; and 16S-R (Primer R), 5′-TACNVGGGTATCT AATCC-3′.

The total PCR reaction system was 18 *μ*L, including 10.0 *μ*L of 2 × Master Mix, 0.5 *μ*L of Primer F (10 *μ*M), 0.5 *μ*L of Primer R (10 *μ*M), and H_2_O up to the total volume of 18 *μ*L. The 18 *μ*L of mixture was added to each well of the 96-well PCR plate, and the corresponding reverse transcription was conducted by adding 2.0 *μ*L of cDNA in each well for each sample. The mixture was briefly centrifuged and placed on ice for short time. Then, PCR reaction was conducted on RT-PCR instrument according to the program including 95°C for 30 s and 40 PCR cycles (including 95°C for 5 s and 60°C for 40 s (fluorescent) collection). In order to establish a PCR product melt curve, after the amplification reaction, according to the designated program including 95°C for 10 s, 60°C for 60 s, and 95°C for 15 s, the mixture was heated slowly from 60°C to 99°C (the Ramp rate was automatically set up as 0.05°C/s). The cDNA sample template was selected for 10-fold gradient dilution; each sample was subjected to PCR determination from 5 dilution gradients in triple for each dilution gradient. The target gene (*nif*H) and internal reference (16S) of each sample were amplified by RT-PCR reaction, respectively. After amplification, the amplification curve, dissolution curve, and corresponding CT value (reaching up to the threshold cycle number) were saved. The data were analyzed by using 2^-△△CT^ method. The calculation formula was △CT = CT_*nif*H_ − CT_16S_; △△CT = △CT_*nif*H gene at different growth stages_ − △CT_*nif*H gene at the seedling stage_ (the gene expression of *nif*H gene at the seedling stage was as control in this experiment). The 2^-△△CT^ represents the relative expression amount of the target gene, which is the change fold for the relative expression of *nif*H gene in each sample at different growth stages when compared with the control at the seedling stage. The measured 2^-△△CT^values≧2 were considered as the criteria for high expression [21].

### 2.5. DNA Extraction and Quantitative Analysis of nifH Gene in Samples

In order to analyze the absolute quantification of *nif*H gene in each sample, the copy number of *nif*H gene in each sample was measured by qRT-PCR. The DNA in all samples was extracted by bacterial genomic DNA extraction reagents according to the product manual. DNA fragments from all samples were detected by 1% agarose gel electrophoresis. The specific gene amplification of *nif*H in each sample was conducted by PCR, and the designed *nif*H gene primers were the same as above. The 50 *μ*L of PCR reaction system included 1.0 *μ*L of Primer F (10 *μ*M), 1.0 *μ*L of Primer R (10 *μ*M), 1.0 *μ*L of DNA template, 25 *μ*L of 2 × Taq MasterMix, and 22 *μ*L of ddH_2_O. PCR reaction was conducted under the conditions: denaturation at 94°C for 5 min, denaturation at 94°C for 30 s, annealing at 55°C for 30 s, and extension at 72°C for 30 s, as well as the amplification cycle of 30 and final extension at 72°C for 10 min. After the reaction, 1% agarose gel electrophoresis was used to evaluate the amplification results, and the target fragment was recovered.

The recovered PCR products were spliced with T carrier, and the spicing system (10 *μ*L) included recovered 4 *μ*L of target fragment,1 *μ*L of T-vector, 5 *μ*L of 2× solution buffer, and splicing at 22°C for approximately 4 h. After transformed into *E. coli* DH5*α*cells, the mixture solution containing transfected cells was applied to the cultivation plate. The positive white colonies were selected for PCR identification. The 10 *μ*L of PCR reaction system included 0.2 *μ*L of Primer F (10 *μ*M), 0.2 *μ*L of Primer R (10 *μ*M), 1.0 *μ*L of DNA template, 5 *μ*L of 2 × Taq MasterMix, and 3.6 *μ*L of ddH_2_O. PCR reaction conditions were the same as above. After the reaction, 1% agarose gel electrophoresis was used to evaluate the amplification results, and plasmids were extracted as the absolute quantitative standard for *nif*H gene in samples.

All DNA samples of each sample were prepared for 18 *μ*L of RT-PCR reaction system, and PCR was performed on RT-PCR instrument according to the same conditions used for the analysis of *nif*H gene. The extracted plasmid was diluted by 10 gradients from 10^1^ to 10^5^, and 2 *μ*L of the PCR amplified product from each dilution gradient was used as the template to establish the standard curve. During fluorescence quantitative detection, 2 *μ*L of diluted DNA was taken as the reaction amount after each sample was diluted by 10 folds, and the determination of each sample was conducted in triple.

### 2.6. Statistical Analysis

The data obtained were analyzed by One-way ANOVA using the SPSS 17.0 software package, and *p* < 0.05 was significant. The Waller-Duncan method was used for multiple comparisons between groups.

## 3. Results

### 3.1. Analysis for the Flora Compositions of Nitrogen-Fixing Bacteria in Roots of P. giganteum at Different Growth Stages

**Figure 1:**
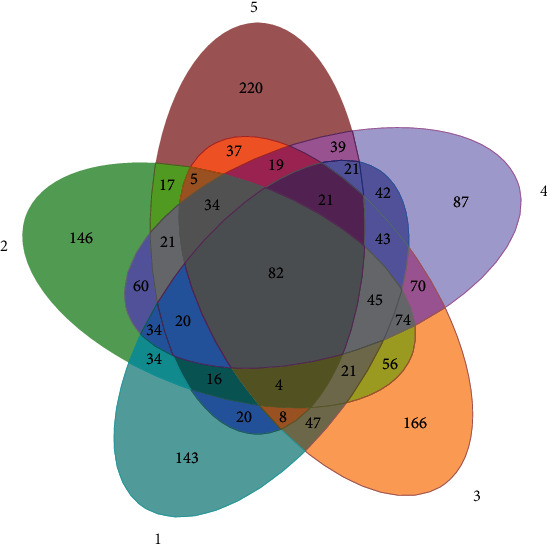
Venn diagram of operational taxonomic unit in 5 samples. The total OTUs (operational taxonomic units) of the 5 samples were 611, 683, 732, 712, and 584, respectively, and shared 82 identical OTUs. Note: (1) seedling stage, (2) tillering stage, (3) jointing stage, (4) maturation stage, and (5) dying stage.

After high-throughput sequencing by Miseq (Illumina) high-throughput sequencing platform, the low-quality sequences and fuzzy sequences were removed. More than 40,000-50,000 effective sequences with completely matched index in 5 samples from *P. giganteum* at different growth stages were obtained, which were mainly distributed around 360 bp. Diversity index statistics of microorganisms and the sequence number of operational taxons in 5 samples are shown in Table 1 and Figure 1, respectively. The results showed that the two evaluation indexes had the same change trend (jointing stage > maturation stage >tillering stage > seedling stage > dying stage), and there were 82 commu-OTUs and 87~220 specific OTUs in five samples, which indicated that there was a certain amount of the same nitrogen-fixing bacteria flora composition in each sample, but the composition of flora varied greatly among the samples. The rarefaction curves of 5 samples were drawn by the Mothur software, and the curves of 5 samples eventually tend to flatten, which indicated that the measured data amount of the samples taken was reasonable and sufficient.

**Figure 2:**
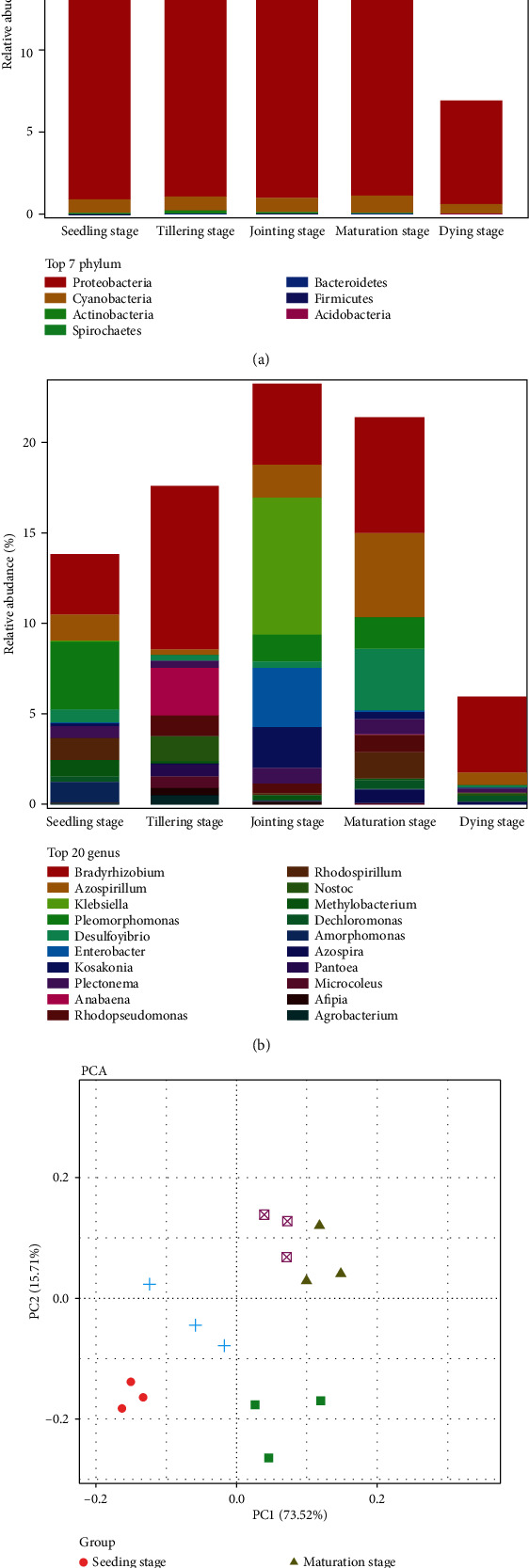
Comparison of the major bacterial composition in 5 samples based on *nif*H sequences. (a) Comparison of flora at the phylum level. Proteobacteria and Cyanobacteria had the highest abundance in each sample. (b) Comparison of flora at the genus level. *Bradyrhizobium* had the higher abundance in each sample. (c) PCA analysis of flora at the genus level. Samples from jointing stage and maturation stage could be better clustered together.

Species annotation was carried out for the OTUs in each sample, as shown in Figure 2. The results showed that there were a large number of endophytic nitrogen-fixing flora of nonclassified species in the roots of *P. giganteum* at different growth stages (between 5.9 and 92.3%), while in the defined species, the predominant species was Proteobacteria and Cyanobacteria (Figure 2(a)), especially, Proteobacteria had the highest abundance (between 6.3 and 24.0%). However, Actinobacteria, Spirochaetes, Bacteroidetes, Firmicutes, and Acidobacteria were distributed in small quantity at different growth periods, and the abundance was less than 0.2%. From the genus level, the changes in species and abundance of endogenous nitrogen-fixing bacteria in roots of *P. giganteum* at different growth stages revealed the increase first and the decrease sequentially (Figure 2(b)), among which the species and abundance of nitrogen-fixing bacteria at the jointing stage and maturation stage were significantly higher than those at other growth stages. PCA analysis showed that the flora at the jointing stage and the maturation stage could be clustered together, and the composition of flora was different from other stages (Figure 2(c)), and the results were consistent with commu-OTU analysis. *Bradyrhizobium* as the core genera was always distributed in each sample and had the higher abundance (3.3-9.0%). In addition, the genus with high abundance at each growth stage also includes *Azospirillum*, *Klebsiella*, *Pleomorphomonas*, and *Desulfovibrio*.

**Figure 3:**
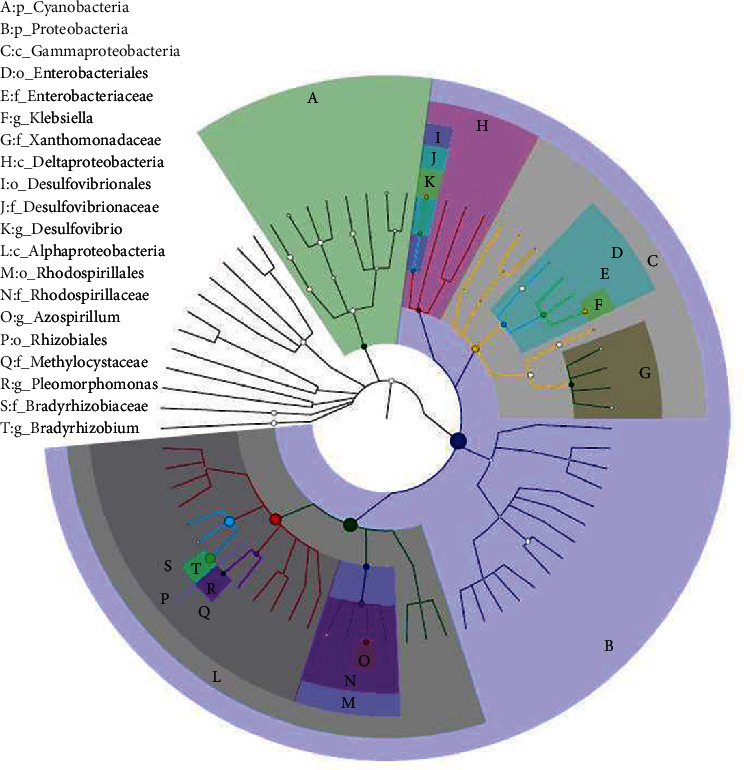
The classification hierarchy tree of entire samples based on the GraPhlAn. The relative abundance ranked among the top 20 was identified by letters in the figure, from phylum level to genus level, arranged from outer layer to inner layer, and the size of the node in the graph represents the average relative abundance ranked. As shown in the picture, from the genus level, the largest node was T g_*Bradyrhizobium*.

The rank tree was drawn based on the overall classification of the GraPhlAn in samples, and the relative abundance ranked among the top 20 is shown in Figure 3. The results showed that Proteobacteria and Cyanobacteria were the phylum with high abundance, and Proteobacteria was the dominant one. *Bradyrhizobium*, *Azospirillum*, *Klebsiella*, *Desulfovibrio*, and *Pleomorphomonas* at the genus level had the high abundance, which was consistent with the above statistical results. *Bradyrhizobium* is the major genus in nitrogen-fixing bacteria from many leguminous plants and gramineous plants (Poly et al. 2001) [19]. The node size in Figure 3 indicated that the abundance of *Bradyrhizobium* was significantly higher than that of other genera of bacteria and was the most important genus of endogenous nitrogen-fixing bacteria in *P. giganteum* at different growth periods.

### 3.2. Analysis of nifH Gene Expression in Roots of P. giganteum at Different Growth Stages

**Figure 4:**
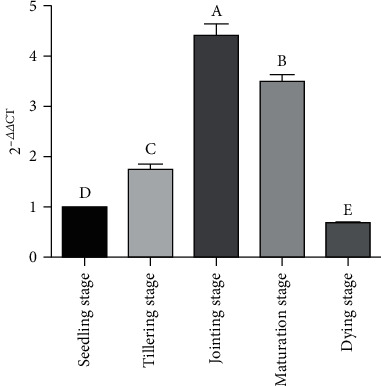
Relative expression value of *nif*H gene at different growth stages. The expression of *nif*H gene at different growth stages exhibited a downward trend after the initial increase. The value of 2^-△△CT^ at jointing stage was the highest, which was 4.43 times as much as at the seedling stage, and the value of 2^-△△CT^ at dying stage was lowest, which was 0.67 times. There were significant differences in 2^-△△CT^ values among groups during each period (*p* < 0.05). The 2^-△△CT^ values in each period make a great difference, and there were significant differences among each group.

1% agarose gel electrophoresis showed that the total RNA extracted from 5 samples had high quality, and the expression of *nif*H gene in 5 samples determined by qRT-PCR; the dissolution curves of each sample revealed the single sharp peak, indicating the single, stable, and reproducible product amplification in each sample and the accurate and reliable results. The amplification efficiency detection of real-time quantitative PCR for *nif*H and 16S gene indicating that the amplification efficiency of the target gene *nif*H was consistent with that of the internal reference gene 16S, and 2^-△△CT^ method could be applied for relative quantitative analysis in the present study. The results showed that the 2^-△△CT^ values of *nif*H gene transcription level in roots of *P. giganteum* at different growth stages were 1 at seedling stage, 1.75 at tillering stage, 4.43 at jointing stage, 3.50 at mature stage, and 0.67 at decay stage (Figure 4), and the change of transcript levels of *nif*H genes exhibited a downward trend after the initial increase, which was consistent with the changing trend of flora compositions of nitrogen-fixing bacteria in roots of *P*. *giganteum* at different growth stages. In addition, the relative expression level of *nif*H gene at the jointing and maturation stages reached the over expression level (≧2), and the relative expression level at the jointing stage was the highest, with significant difference compared with seedling stage (*p* < 0.05).

### 3.3. The Analysis of nifH Gene Copy Number in Roots of P. giganteum at Different Growth Stages

**Figure 5:**
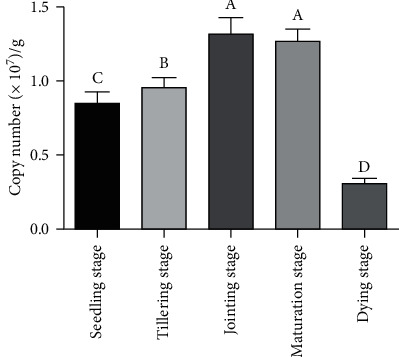
Quantitative result of *nif*H gene at different growth stages. The copy number of *nif*H gene at different growth stages revealed an initial increase and a sequential decrease. The *nif*H gene copy number of 1.32 × 10^7^/g at jointing stage was highest; while dying stage was lowest, the copy number was 0.31 × 10^7^/g.

1% agarose gel electrophoresis showed that the total DNA extracted from 5 samples had high quality, and the qRT-PCR amplification curves of the extracted plasmid standard substance and 5 samples and the dissolution curves of the amplified products showed that the amplified products were single, stable, and reproducible, which could be used for further analysis. The copy number of *nif*H gene in roots of *P. giganteum* at different growth stages was determined by qRT-PCR (Figure 5). The results showed that the copy number of *nif*H gene in roots of *P. giganteum* at different growth stages were 0.85 × 10^7^/g at seedling stage, 0.96 × 10^7^/g at tillering stage, 1.32 × 10^7^/g at jointing stage, 1.27 × 10^7^/g at mature stage, and 0.31 × 10^7^/g at decay stage, and the change of copy number of *nif*H gene exhibited an initial increase and a sequential decrease, which was consistent with the aforementioned changing trend of the flora compositions in nitrogen-fixing bacteria and the relative expression of *nif*H gene. The copy number of *nif*H gene in roots of *P. giganteum* at different growth stages revealed the significant difference except the jointing stage and maturation stage (*p* < 0.05).

## 4. Discussion

Many gramineous plants, such as maize, rice, sugarcane, and herbage, have a large number of endogenous nitrogen-fixing bacteria [22]. The *nif*H gene is the major nitrogen-fixing gene in endogenous nitrogen-fixing bacteria and is also the major gene for scholars to investigate the combinatorial nitrogen-fixing function of gramineous plants [23, 24]. It is generally believed that the more microbial species in a sample could result in the more individual population and the more copies of functional genes [25]. In this study, high-throughput sequencing showed that the flora composition of nitrogen-fixing bacteria was the highest at the jointing stage and maturation stage, while the expression level and copy number of *nif*H gene in nitrogen-fixing bacteria determined by qRT-PCR were the highest, which revealed the consistent rules.

Nitrogen is one of the indispensable nutrient elements for the growth and development of crops. During the process of combinatorial nitrogen fixation, endophytic nitrogen-fixing bacteria occupy an important ecological niche, which can perform combinatorial nitrogen fixation in the rhizosphere or *in vivo*, thus providing nitrogen or growth-promoting substances for plant growth [26, 27]. The gramineous plants grow from the seedling stage to the tillering stage, and its endogenous nitrogen-fixing bacteria gradually become active, and the nitrogen-fixing gene nifH genes then begin to be expressed, and the copy number increases, thereby providing certain nitrogen for plant growth. The jointing stage is the rapid growth period of gramineous plants. Compared with other growth stages, endogenous nitrogen-fixing bacteria at the jointing stage grow extremely, with a large number of species and total bacteria, and the expression amount and copy number of nitrogen-fixing gene *nif*H reached up to the maximum level. Then, the growth of mature plants is completed, and the species of nitrogen-fixing bacteria and the expression of *nif*H gene tend to the stable level and then the slow decline finally. During the dying stage, plant growth stops, and nutrients are exhausted; the endogenous nitrogen-fixing bacteria in plants reveal the rapid death, the species and total amount of flora decrease, and the expression amount and copy number of *nif*H gene decrease to the lowest level.

In conclusion, the flora compositions of endogenous nitrogen-fixing bacteria and the expression amount and copy number of nitrogen-fixing gene *nif*H in plants were closely related to plant growth. At the jointing stage, the roots of gramineous plants contain several major groups of combinatorial nitrogen-fixing bacteria [28], such as *Bradyrhizobium*, *Azospirillum*, and *Klebsiella*, with high abundance, which will be an important source of endogenous nitrogen-fixing bacteria to isolate specific nitrogen-fixing bacteria from *P. giganteum*. This study can be used as the guidance for further developing cultivable and potential endogenous azotobacter resources from *P. giganteum* and can be establish an endogenous azotobacter germplasm repository from gramineous plants to explore the combinatorial nitrogen-fixing mechanism, thereby providing the fundamental direction for the development and utilization of azotobacter products as farm microbial agents [29].

## 5. Conclusion

In the present study, high-throughput sequencing technology was used to determine the flora compositions of endogenous nitrogen-fixing bacteria in roots of *P. giganteum* at different growth stages. According to the number of OTU sequences and the species and abundance of nitrogen-fixing bacteria at different classification levels, the change of bacteria revealed an order as jointing stage > maturation stage >tillering stage > seedling stage >dying stage. Proteobacteria and Cyanobacteria were the major phylum of nitrogen-fixing bacteria, and *Bradyrhizobium* was the core genera. The qRT-PCR assays of each sample showed that the changing trend of relative expression and quantitative of *nif*H gene in nitrogen-fixing bacteria from roots of *P. giganteum* at different growth stages was consistent with the change in flora compositions of nitrogen-fixing bacteria, and both showed a trend of an initial increase and a sequential decrease. The relative expression level and copy number of *nif*H gene in each stage were significantly different among different groups (*p* < 0.05), and the relative expression level and copy number were the highest at the jointing stage, which was 4.43 times of that at the seedling stage determined by 2^-△△CT^ method, and the copy number was 1.32 × 10^7^/g. While at dying stage, it was the lowest, and the 2^-△△CT^ value was 0.67 folds, with the copy number of 0.31 × 10^7^/g.

## Figures and Tables

**Table 1:** The sequencing data and diversity index statistics in 5 samples.

Target	Samples	Number of valid sequence	Diversity indices			
			Simpson	Chao1	ACE	Shannon
*nifH*	Seeding stage	42113 ± 563^ab^	0.98 ± 0.02^ab^	625.24 ± 12.36^c^	628.98 ± 11.12^c^	6.71 ± 0.43^b^
	Tillering stage	40689 ± 231^c^	0.97 ± 0.01^ab^	693.52 ± 10.23^b^	686.29 ± 13.23^b^	6.85 ± 0.58^b^
	Jointing stage	46890 ± 612^a^	0.99 ± 0.01^a^	759.88 ± 22.12^a^	739.99 ± 14.42^a^	7.73 ± 0.48^a^
	Mature stage	41436 ± 226^b^	0.97 ± 0.02^ab^	732.00 ± 23.12^ab^	732.00 ± 13.23^ab^	6.85 ± 0.66^b^
	Dying stage	40332 ± 253^c^	0.96 ± 0.02^b^	598.72 ± 10.12^c^	598.65 ± 10.78^c^	6.46 ± 0.41^c^

The data were expressed as mean ± standard deviation, and the Duncan method was used for multiple comparisons between groups; the same letter in the same column means it has no significant difference (*p* > 0.05), and the different letter means it has significant difference (*p* < 0.05).

## Data Availability

All data are fully available without restriction, and all relevant data are within the paper.
